# Transcriptomic Profiling and Tumor Microenvironment Classification Reveal Unique and Dynamic Immune Biology in HIV-Associated Kaposi Sarcoma

**DOI:** 10.3390/cells14020134

**Published:** 2025-01-17

**Authors:** Jihua Yang, Ayse Ece Cali Daylan, Aleksei Shevkoplias, Ekaterina Postovalova, Meng Wang, Andrey Tyshevich, Matthew Lee, Hiba Narvel, Ksenia Zornikova, Nara Shin, Nikita Kotlov, Luca Paoluzzi, Changcheng Zhu, Balazs Halmos, Xingxing Zang, Haiying Cheng

**Affiliations:** 1 Department of Oncology (Medical Oncology), Montefiore Medical Center, Albert Einstein College of Medicine, Bronx, NY 10461, USA; jihua.yang@einsteinmed.edu (J.Y.); ayseece@wustl.edu (A.E.C.D.); meng.wang@einsteinmed.edu (M.W.); mlee7@montefiore.org (M.L.); bahalmos@montefiore.org (B.H.); xingxing.zang@einsteinmed.edu (X.Z.); 2 Research and Development, BostonGene Corporation, Waltham, MA 02453, USA; alexey.shevkoplyas@bostongene.com (A.S.); ekaterina.postovalova@bostongene.com (E.P.); ksenia.zornikova@bostongene.com (K.Z.); nikita.kotlov@bostongene.com (N.K.); 3 Department of Medicine, Jacobi Medical Center, Bronx, NY 10461, USA; narvelhiba@gmail.com; 4 Clinical Sciences, Oncology, Regeneron Pharmaceuticals Inc., Tarrytown, NY 10591, USA; lucapaoluzzi@gmail.com; 5 Department of Pathology, Montefiore Medical Center, Albert Einstein College of Medicine, Bronx, NY 10461, USA; czhu@montefiore.org

**Keywords:** Kaposi sarcoma, immune milieu, transcriptomics, TCR clonotype, tumor microenvironment, gene signatures, HIV

## Abstract

Kaposi Sarcoma (KS) is a vascular tumor originating from endothelial cells and is associated with human herpesvirus 8 (KSHV) infection. It disproportionately affects populations facing health disparities. Although antiretroviral therapy (ART) has improved KS control in people with HIV (PWH), treatment options for advanced KS remain limited. This study investigates the tumor microenvironment (TME) of KS through whole-transcriptomic profiling, analyzing changes over time and differences based on HIV status. The TME was categorized into four subtypes: immune-enriched (IE), non-fibrotic, immune-enriched/fibrotic (IE/F), fibrotic (F) and immune-depleted (D). Nine KS patients (four HIV-negative and five HIV-positive) were enrolled in the study. Longitudinally collected KS samples from three patients (one HIV-negative and two HIV-positive) allowed for the investigation of dynamic TME changes within individual patients. The immune cellular composition was determined using deconvolution and compared to a cohort of non-KS patients. Our findings revealed that all KS samples, regardless of HIV status, were enriched in endothelial cells. Compared to non-KS tissues, the KS samples contained a higher percentage of NK and CD8+ T cells. HIV-negative KS samples displayed the IE and IE/F TME subtypes, while HIV-positive samples exhibited IE, IE/F, and F subtypes. Over the course of the disease, a decrease in angiogenic signatures was observed in two HIV-positive KS patients. Notably, HIV-negative KS samples showed alterations in NK cell-mediated immunity and cytotoxic response pathways, whereas HIV-positive samples exhibited changes in growth regulation and protein kinase activity pathways at the time of initial diagnosis. The gene expression of immune checkpoints, including CD274 (PD-L1) and PDCD1LC2 (PD-L2), was comparable between HIV-positive and HIV-negative KS samples at diagnosis. Furthermore, sequencing identified a shared TCR*β* chain in all patients analyzed, indicating a T-cell immune response to a common antigen. This study demonstrates unique transcriptomic features and TME subtypes in KS that differ based on HIV status. Additionally, it illustrates longitudinal dynamic changes in the gene signatures and TME subtypes in individual patients. The identification of a shared TCRβ chain suggests that immune T cells in KS patients may target a common antigen. Future studies should further explore the immune microenvironment and unique T cell clonotypes, which could pave the way for the development of novel therapeutic strategies for KS patients.

## 1. Background

Kaposi sarcoma (KS) is a rare vascular malignancy associated with human herpesvirus-8 (HHV-8/KSHV). It often arises in immunosuppressed individuals, particularly people with HIV (PWH). KS was one of the hallmark cancers of the AIDS epidemic, and despite advancements in antiretroviral therapy (ART), disease control remains a significant challenge for many patients [1]. In 2020, it was estimated that over 34,000 new KS cases and over 15,000 KS-related deaths occurred globally, with the majority of the cases occurring in Southern and Eastern Africa [2]. While KS is classified as an AIDS-defining cancer, it can also affect patients without HIV. Based on clinical and histological features, KS can be categorized into four clinical subtypes: (1) the classic or sporadic subtype, characterized by indolent growth, which is typically seen in Mediterranean countries; (2) the epidemic subtype, which is observed in PWH; (3) the endemic subtype, which is seen in Sub-Saharan countries; and (4) the iatrogenic subtype, which occurs in patients receiving immunosuppressive therapy, such as organ transplant patients [3].

Since the introduction of effective ART in 1996, the incidence of KS in PWH has declined significantly [1]. However, KS remains a significant public health concern in low-income countries with a high HIV burden, highlighting the critical need to address global healthcare disparities [4]. Despite the availability of highly effective antiretroviral therapies, PWH continue to face significantly elevated risk of KS, with a standard incidence ratio of 35 compared to the general population [5].

Management options for KS depend on its subtype. For epidemic and iatrogenic KS, treatment often begins with ART and a reduction in immunosuppression, respectively. However, when the above strategies become insufficient, or in cases of classic or endemic KS, therapeutic options are limited [1]. Chemotherapy is commonly used, and a few clinical trials using immunomodulators, immunotherapy agents, and targeted therapies are currently underway [6].

Immune evasion is recognized as one of the eight hallmarks of cancer [7]. The tumor microenvironment (TME) comprises a heterogeneous mix of cells, including infiltrating immune and stromal cells, which plays various roles in cancer progression. The TME can either suppress or promote tumor growth. On the one hand, it facilitates cytotoxicity by secreting cytokines, recruiting effector cells, and presenting tumor antigens. On the other hand, it can drive tumor progression by promoting angiogenesis and metastasis and inhibiting effective cytotoxic T cell responses [8]. TME subtypes are correlated with clinical outcomes and immunotherapy responses [9]. A prior study identified four TME subtypes across twenty different cancers based on gene signatures: immune-enriched/fibrotic (IE/F), immune-enriched/non-fibrotic (IE), fibrotic (F) and immune-depleted (D). This study highlighted the potential of multiparametric biomarkers, such as TME composition, in predicting immunotherapy efficacy. Notably, TME subtyping outperformed many traditional predictive biomarkers in predicting immunotherapy outcomes across different cancer subtypes [10].

Characterization of the TME in HIV-associated cancers is particularly critical, as HIV infection and antiretroviral therapies are known to alter immune cells and their interactions with cancer cells [11,12]. Prior studies have identified differences in the TME of HIV-associated cancers compared to cancers in the general population [13]. For instance, our group recently showed that NSCLC in PWH had low CD8+ tumor-infiltrating lymphocytes counts and limited PD-L1 expression compared to the NSCLC in the general population [14]. A better understanding of the KS immune milieu, especially its relationship with HIV status and its dynamic nature within individual patients, is essential, as it could inform therapeutic strategies. This study aims to investigate the TME of KS through transcriptomic analysis of HIV-positive and HIV-negative samples, with a focus on its dynamic nature and potential implications for treatment.

## 2. Methods

### 2.1. Patients

Nine patients with pathologically confirmed KS were enrolled in this study. Three of the patients (two HIV-positive and one HIV-negative) had multiple samples collected longitudinally to study the dynamic changes in TME in individual patients. The specimens were collected from the skin or other sites of metastases at the treating physicians’ discretion. The clinicopathological data, including demographics, HIV disease characteristics, and treatment history, were collected through a retrospective chart review. All protocols were reviewed and approved by the Institutional Review Board of Montefiore Medical Center/Albert Einstein College of Medicine. The meta-cohort of non-KS patients (*n* = 1046) was provided by BostonGene, Boston, MA, USA.

### 2.2. Bulk RNA Sequencing (RNAseq)

Whole-transcriptomic profiling was performed on thirteen retrospective tissue samples from nine patients. RNA from FFPE specimens was isolated using the AllPrep DNA/RNA FFPE Kit (Cat. 80234, Qiagen, Hilden, Germany) as per the manufacturer’s protocol. Next, 20 to 200 ng of total RNA from RNA-Seq libraries was prepared using the Agilent XT HS2 RNA Kit with V7+UTR probes (Agilent, Santa Clara, CA, USA). All library preparations were performed as per the manufacturer’s instructions. Cleanup procedures were performed using Agencourt AMPure XP beads (Beckman Coulter, Brea, CA, USA, Cat A63881). All RNA libraries were sequenced on NovaSeq 6000 (Illumina, San Diego, CA, USA). The samples were sequenced with a target of 50 M reads using 151 bp paired-end sequencing.

### 2.3. NGS Data Quality Control

FastQC v0.11.5, FastQ Screen v0.11.1, RSeQC v3.0.0 and MultiQC v1.6 were used to perform quality control (QC) of all NGS samples. Sample correspondence was checked by HLA comparison from RNA-Seq using OptiType70.

### 2.4. Deconvolution

The novel machine learning algorithm, Kassandra, was used to predict the cell percentages from bulk RNA-seq [15]. The model consisted of a two-level hierarchical ensemble that used LightGBM as building blocks. The model was trained on artificial RNA-seq mixtures of different cell types (T cells, B cells, NK, macrophages, cancer-associated fibroblasts, and endothelial cells), obtained from multiple datasets of sorted cells. All datasets were isolated from poly-A or total RNA-seq profiled human tissues with read lengths higher than 31 bp and at least 4 million coding read counts. These datasets passed quality control via FASTQC with minimal contamination (<2%). The model was trained to predict the percentage of RNA belonging to specific cell types. Predicted percentages of RNA were later converted into percentages of cells using the previously described methodology [16].

### 2.5. Gene Expression Signatures and TME

RNA-sequencing reads were aligned with GENCODE v23 transcripts using Kallisto v0.42.4 with default parameters. Retained transcripts included protein-coding transcripts as well as immunoglobulin-heavy kappa and lambda light chains, and TCR-related transcripts. Noncoding RNA, histone, and mitochondria-related transcripts were excluded, resulting in 20,062 protein-coding genes. Gene expression levels were quantified as transcripts per million (TPM) and subsequently log2-transformed [17]. Gene signature scores were derived using the ssGSEA algorithm implemented in custom script (https://github.com/BostonGene/MFP/, accessed on 1 February 2023). Differential gene expression analysis was performed by EdgeR and TME types were estimated according to the prevMFP method.

### 2.6. RNA-Seq Data Processing and Differential Expression Analysis

Gene expression data were quantified as Transcripts Per Million (TPM). Genes with consistently low expression across all samples were filtered out to reduce noise. Differential expression analysis between HIV-positive (HIV+) and HIV-negative (HIV−) KS samples was performed using a two-sample independent *t*-test on log2-transformed TPM values. The log2 fold-change (log2FC) was calculated as the difference in mean expression between HIV+ and HIV− samples. Genes with *p* < 0.05 and |log2FC| > 1 were considered statistically significant.

### 2.7. Volcano Plot

The volcano plot was generated to visualize differential expression results. The *x*-axis represents log2 fold-change (HIV+ vs. HIV−) and the *y*-axis represents −log10 (*p*-value). All analyzed genes are shown, with statistically significant genes highlighted in purple (upregulated in HIV+) and orange (upregulated in HIV−). The most significant genes, based on *p*-value, were labeled for clarity.

### 2.8. Gene Ontology (GO) Enrichment Analysis

Gene Ontology (GO) enrichment analysis was performed separately for significantly upregulated genes in HIV+ and HIV− KS samples. Enrichment was assessed using the gseapy Python library with the “GO Biological Process 2021” database. A ranked list of upregulated genes (based on log2FC) was analyzed to identify overrepresented biological processes. Adjusted *p*-values (FDR) were used to rank the top enriched terms, which were visualized as bar plots.

### 2.9. Principal Component Analysis (PCA)

PCA was conducted to assess overall variation in gene expression across samples. The analysis was performed on the top 1000 most variable genes (based on standard deviation of TPM values) to capture the most biologically relevant differences. Gene expression data were standardized using z-score normalization prior to PCA. The first two principal components (PC1 and PC2) were visualized, showing clear separation between HIV+ (purple) and HIV− (orange) samples.

### 2.10. Identification of Top Genes Contributing to PCs

Individual genes’ contributions to PC1 and PC2 were determined using PCA loadings. Loadings represent the coefficients for each gene in the linear combination defining a principal component. Genes with the highest absolute loadings for PC1 and PC2 were identified and ranked. The top contributing genes were visualized as horizontal bar plots.

### 2.11. T-Cell Receptor (TCR) Analysis

Extraction of data for TCR clonotypes from raw FASTQ files was executed with MiXCR version 3.0.12 [18] with default parameters for bulk RNA-seq extraction. The TCR logo was plotted using “ggseqlogo” package version 0.1.

### 2.12. KSHV Status

KSHV viral reads identification was assessed with the GATK 4 Pathseq software kit [19] with quantitative assessment expressed in viral reads per million human reads (VRM), and their viral status was verified with VIRTUS.

## 3. Results

### 3.1. Baseline Characteristics of KS Patients

Nine KS patients were included in this study (Table 1). Of these, four were HIV-negative and five were HIV-positive. To investigate temporal changes in TME, we analyzed multiple samples collected at different time points from one of the HIV-negative and two of the HIV-positive KS patients. All patients were male, with a median age of 37 years (range 32–80). The majority of patients had an ECOG performance status (PS) of 0–1, except for one patient with an ECOG PS of 3. Three patients presented with metastatic disease, and five were categorized as poor-risk. Two patients had CD4 cell counts <100 cells/μL. Most patients received local therapies, while three underwent systemic chemotherapy consisting of liposomal doxorubicin (Table 1).

#### Immune Cellular Deconvolution of KS Specimens

**Figure 1:**
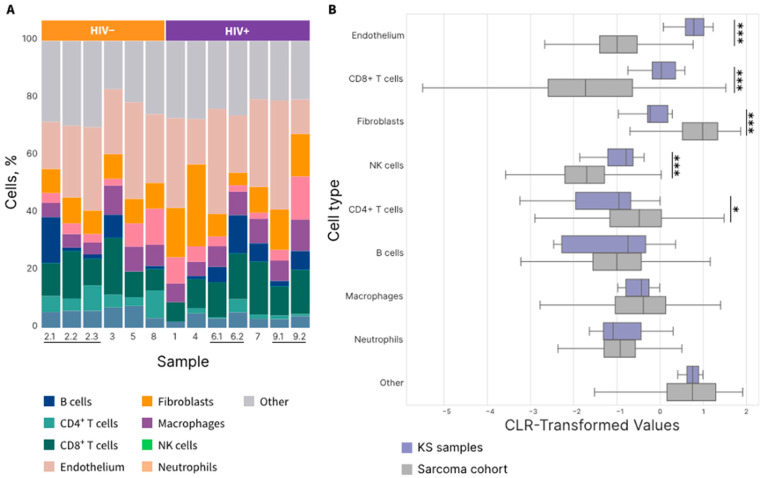
(**A**) Cellular composition of Kaposi’s sarcoma (KS) samples (n = 13), as determined using the Kassandra deconvolution algorithm. Connected lines at the bottom represent sequential samples collected from the same patient, with sample IDs indicating collection order (e.g., 2.1, 2.2, 2.3 represent sequential samples from patient 2). (**B**) Comparative analysis of cell type proportions between KS samples (n = 13) and BostonGene’s internal sarcoma cohort (n = 1046), shown using center log ratio (CLR)-transformed values. The analysis includes all cell types predicted by the deconvolution algorithm. Bars represent means with confidence intervals. * *p* < 0.05, *** *p* < 0.001. The lines at the bottom indicate sequential samples from the same patient.

The immune cell distribution of the KS specimens was analyzed using the Kassandra deconvolution algorithm of the transcriptome (Figure 1A). The KS cohort had a higher proportion of endothelial, CD4+ and CD8+ cells, alongside a lower proportion of fibroblasts compared to the meta-cohort of non-KS samples (Figure 1B). The majority of the non-KS meta-cohort consisted of patients with leiomyosarcoma (40%) and liposarcoma (22%). Notably, macrophages were abundant in KS TME irrespective of the HIV status.

### 3.2. TME Subtypes in KS Specimens Based on Gene Expression Profiles

**Figure 2:**
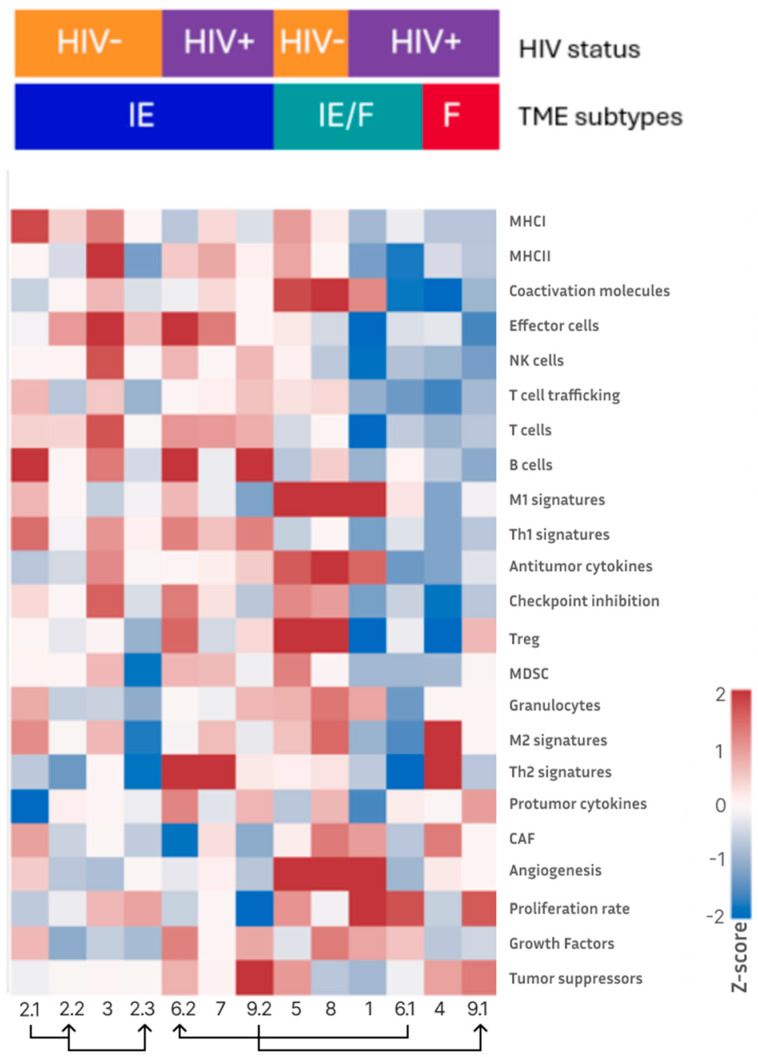
TME subtypes identified in the KS cohort. The heatmap shows the normalized ssGSEA score changes in the KS cohort compared to the meta-cohort (z-score scale shown on the right). The top annotation bars indicate HIV status and TME subtypes. The rows represent immune infiltration signatures including MHC expression, immune cell populations (T cells, B cells, NK cells), immune response markers (cytokines, checkpoint molecules), and stromal elements (CAF, angiogenesis). Connected lines and arrows at the bottom indicate sequential samples from the same patient, with arrows pointing in the order of sample collection (e.g., 2.1 → 2.2 → 2.3).

In our KS cohort, three TME subtypes were identified: IE, IE/F, and F (Figure 2). HIV-negative KS samples demonstrated the IE and IE/F TME subtypes. The IE TME subtype was characterized by high levels of immune infiltration and low prevalence of stromal and fibrotic elements (Supplementary Figure S1). The IE/F TME subtype displayed moderate immune infiltration, a high prevalence of stromal and fibrotic elements, along with intense vascularization. HIV-positive KS specimens exhibited all three subtypes: IE, IE/F, and F. The F TME subtype was characterized by minimal immune infiltration, a high prevalence of stromal elements, abundant cancer-associated fibroblasts and moderate angiogenesis. Notably, the D TME subtype, which was observed in other non-KS samples, was absent in our KS cohort.

### 3.3. Comparison of Gene Expression Profiles Between KS Specimens Based on HIV Status

**Figure 3:**
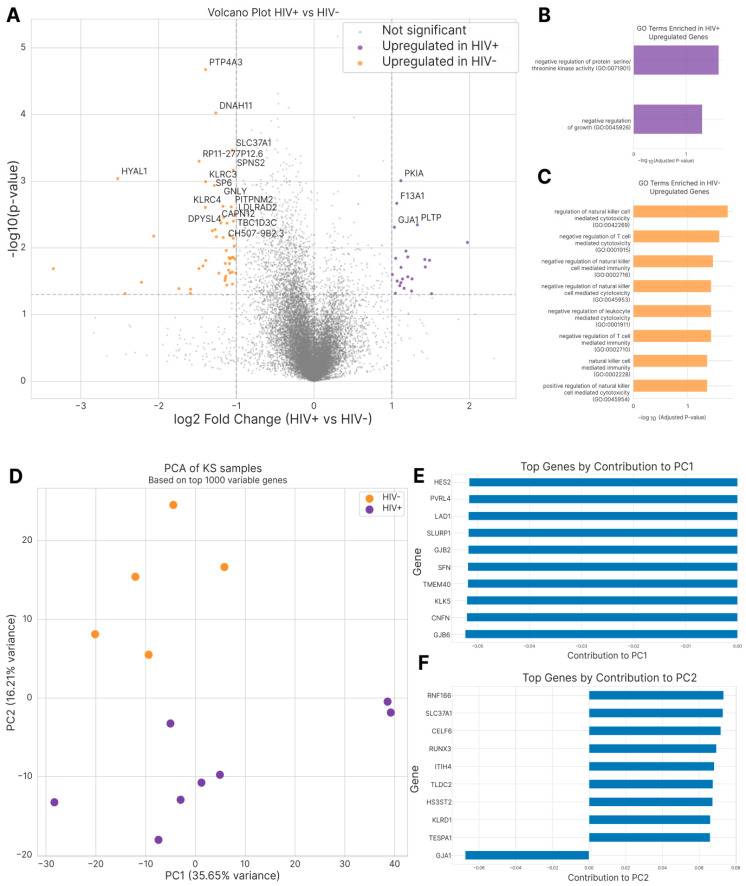
Differential gene expression and pathway analysis between HIV+ and HIV− KS samples. (**A**) Volcano plot showing log2 fold-change (HIV+ vs. HIV−) against −log10(*p*-value) for all genes. Significant genes (*p* < 0.05, |log2FC| > 1) are highlighted in purple (upregulated in HIV+) and orange (upregulated in HIV−), with the top genes labeled. (**B**) Gene Ontology (GO) terms enriched in HIV+ upregulated genes. (**C**) GO terms enriched in HIV− upregulated genes. (**D**) Principal component analysis (PCA) of KS samples based on the top 1000 variable genes, showing clear separation between HIV+ (purple) and HIV− (orange) samples. (**E**) Top genes contributing to PC1, ranked by absolute loading values. (**F**) Top genes contributing to PC2, ranked by absolute loading values.

Distinct differences in gene expression patterns were observed between HIV-negative and HIV-positive KS specimens when the nine initial patient samples were analyzed, as seen in the volcano plot and the principal component analysis (Figure 3). HIV-negative samples showed an enrichment of genes associated with NK cell-mediated immunity and cytotoxic response pathways. In contrast, HIV-positive samples exhibited enrichment of genes related to growth regulation and protein kinase activity pathways. The expression levels of immune checkpoint genes, including *CD274* (PD-L1) and *PDCD1LG2* (PD-L2), did not differ significantly between the two cohorts. Notably, two HIV-positive KS patients (patient #6 and patient #9) with longitudinal paired samples showed a shift towards a less angiogenic TME during the course of their disease.

### 3.4. T-Cell Receptor–Beta (TCR-β) Repertoire Analysis in KS Tumor-Infiltrating Lymphocytes

**Figure 4:**
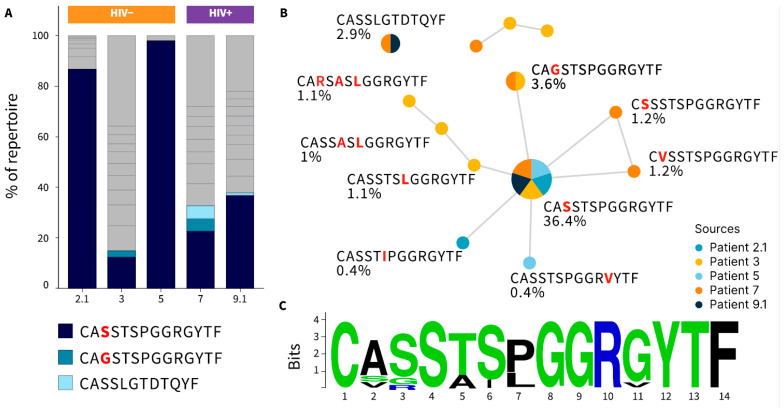
Clonal composition of the TCRβ repertoire of tumor-infiltrating lymphocytes (TILs) was assessed in the KS patients. (**A**) TCR repertoire composition. Shared CDR3β regions are colored. (**B**) Homologous (Hamming distance = 1) and shared CDR3β found in KS samples (substitutions highlighted in red). The median frequency of each clonotype in total TIL repertoire is indicated. (**C**) Position Weight Matrix from the biggest cluster.

We analyzed TCR-β chains in tumor-infiltrating lymphocytes from three HIV-negative KS patients and two HIV-positive KS patients (Figure 4). Three shared TCR-β chains were detected across different patients. Notably, one of these TCR-β chains was found in all five patients, comprising 12–98% of their respective TCR-β repertoires. Additionally, we identified seven highly homologous TCR-β chains within the KS cohort. None of these TCR-β chains were found in VDJdb, a curated database that stores TCR sequences with known antigen specificities [20]. Among patients with shared T cell clonotypes, patients #2 and #5 carried the HLA-A*02, patient #9 had HLA-A*24, and patient #3 had both the HLA-A*02 and HLA-A*24 alleles. These findings suggest a T cell immune response targeting a common antigen, potentially originating from the tumor or KSHV, in these patients.

## 4. Discussion

First described by Moritz Kaposi in 1872, KS is a multicentric endothelial cell cancer that affects the skin, mucosa, lymph nodes and various organs, including the gastrointestinal tract, lungs, and bones [1]. While the incidence of KS has markedly decreased with the early implementation of ART, it remains a significant contributor to long-term morbidity and mortality, especially in populations facing health disparities. Characterizing the immune milieu and altered cellular pathways in KS can be instrumental in developing novel therapeutic strategies to improve patient outcomes.

Our study provides novel insights into the TME of KS, highlighting differences based on HIV status. Macrophages are the predominant tumor-infiltrating immune cells in sarcomas [21], and studies investigating the KS immune microenvironment remain limited. An earlier study employing immunohistochemical staining of PD-L1, CD3, CD33, CD68, and CD168 revealed that KS samples were rich in CD68+/CD163+ macrophages, and CD33+ myeloid-derived suppressor cells [22].

Similarly, in our KS cohort, macrophages represented a substantial proportion of infiltrating immune cells. Compared to the non-KS cohort, our KS cohort demonstrated a higher percentage of endothelial cells—consistent with the endothelial origin of KS—and a lower percentage of fibroblasts. Moreover, more than half (seven out of thirteen samples; five out of nine patients) were categorized as the immune-enriched/non-fibrotic (IE) subtype. The F TME subtype was observed only in two samples, both from HIV-positive patients. Overall, the TME categorization of our KS cohort is notably distinct from the non-KS cohort: Only 18.66% of the non-KS cohort were categorized as IE. In addition, none of the KS sample exhibited a D TME subtype, whereas the D subtype was seen in 17.1% of the non-KS cohort.

Transcriptomic differences based on HIV status were also noted. Lidenge et al. previously compared transcriptome profiles of endemic and epidemic KS lesions, finding that while KSHV is the principal causative factor in both subtypes, HIV-positive KS cases exhibit more pronounced gene dysregulation in pathways affecting tumorigenesis and inflammation/immune responses [23]. Similarly, in our study, HIV-positive KS samples showed enrichment of genes related to protein kinase pathways, while HIV-negative KS samples demonstrated enrichment of genes involved in innate immunity and cytotoxicity pathways. These findings underscore the impact of HIV status on KS pathobiology and TME characteristics.

Our study also revealed that TME features are dynamic. In two HIV-positive KS patients, a longitudinal analysis revealed a shift towards a less angiogenic TME over time. A transcriptomic analysis of KS tumors by another group also highlighted the critical role of angiogenesis pathways in the pathogenesis of this malignancy [24].

Additionally, we identified highly homologous TCR-β chains among KS samples in our cohort, suggesting a T cell response to a common antigen, which was potentially derived from the tumor or KSHV infection. Future studies should investigate potential shared antigens and their interactions with MHC molecules. An improved understanding of TCR clonotypes may provide a foundation for designing T cell-based immunotherapies for KS patients.

Our study is among the few in the literature that aim to provide new insights into the pathogenesis of KS by investigating its TME. However, there are some limitations. The small sample size is a key constraint, though this is expected given the rarity of this disease. While we observed longitudinal changes in the TME, the underlying drivers of these changes remain unclear due to the limited number of paired samples and the variability in the treatments received. It is also important to note that the immune cell composition in HIV-positive KS cases may be influenced by the degree of HIV control and the low CD4 counts. In addition, our analysis relies on transcriptomic signatures, which, while informative, require validation through complementary techniques such as immunohistochemical analysis. Future studies should build on these findings to deepen our understanding of KS pathogenesis.

## 5. Conclusions

Our study investigates the TME of HIV-negative and HIV-positive KS patients using transcriptomic signatures. It provides unique insights into the KS TME by comparing signatures in HIV-positive and -negative cohorts, while also highlighting the dynamic nature of the TME through paired sample analyses. Our findings reveal a significant proportion of the IE TME subtype in KS patients, emphasizing the need for future studies to investigate the potential role of new immunotherapies in this cohort. Importantly, we showed that KS samples, irrespective of HIV status, do not exhibit an immune desert (D) TME type. However, HIV-positive samples can be categorized into both F and IE/F TME subtypes in addition to IE. This suggests that therapeutic strategies targeting the remodeling of fibrotic, immune-suppressive TMEs should be prioritized in HIV-positive KS cases. Finally, the shared T-cell clonotype identified in our cohort warrants further investigation to determine its potential therapeutic implications. Future studies should focus on exploring these shared clonotypes and their interactions, which may ultimately inform the development of T cell-based immunotherapies for KS patients.

## Figures and Tables

**Table 1:** Baseline patient characteristics. ART: antiretroviral therapy; NA: not available; PS: performance status.

Patient	Number of Samples	HIV Status	Age	Sex	Race/Ethnicity	ECOG PS	Stage	Treatment Received	Baseline CD4 Count (Cells/μL)	HIV Viral Load (Copies/mL)
1	1	Positive	32	Male	Hispanic	0	T0 I1 S0	ART	37	<40
2	3	Negative	42	Male	Black	0	T0 I0 S0	Cryotherapy, radiation therapy, resection		
3	1	Negative	73	Male	Hispanic	1	T0 I0 S0	Resection		
4	1	Positive	37	Male	NA	1	T0 I0 S0	ART	377	0
5	1	Negative	80	Male	Hispanic	3	T0 I0 S1	Cryotherapy, radiation therapy		
6	2	Positive	36	Male	Hispanic	1	T1 I1 S1	ART, Radiation therapy, liposomal doxorubicin	96	<40
7	1	Positive	37	Male	Black	1	T1 I0 S1	ART, liposomal doxorubicin	553	0
8	1	Negative	80	Male	Hispanic	1	T0 I0 S0	Radiation therapy		
9	2	Positive	36	Male	Hispanic	1	T1 I0 S1	ART, liposomal doxorubicin	477	121103

## Data Availability

The supporting data are not publicly available due to research participant privacy restrictions.

## Supplementary Materials

The following supporting information can be downloaded at https://www.mdpi.com/article/10.3390/cells14020134/s1. Supplementary Figure S1. Comparison of gene signatures between KS and non-KS sarcoma cohort.
