# Supplementary material for: Transcriptomic Profiling and Tumor Microenvironment Classification Reveal Unique and Dynamic Immune Biology in HIV-Associated Kaposi Sarcoma
Source: Cells. 2025 Jan 17;14(2):134. doi: 10.3390/cells14020134 (PMC11764145; doi:10.3390/cells14020134)
Supplement: Supplementary file 1 [file cells-14-00134-s001.zip › cells-3286168-supplementary.pdf]

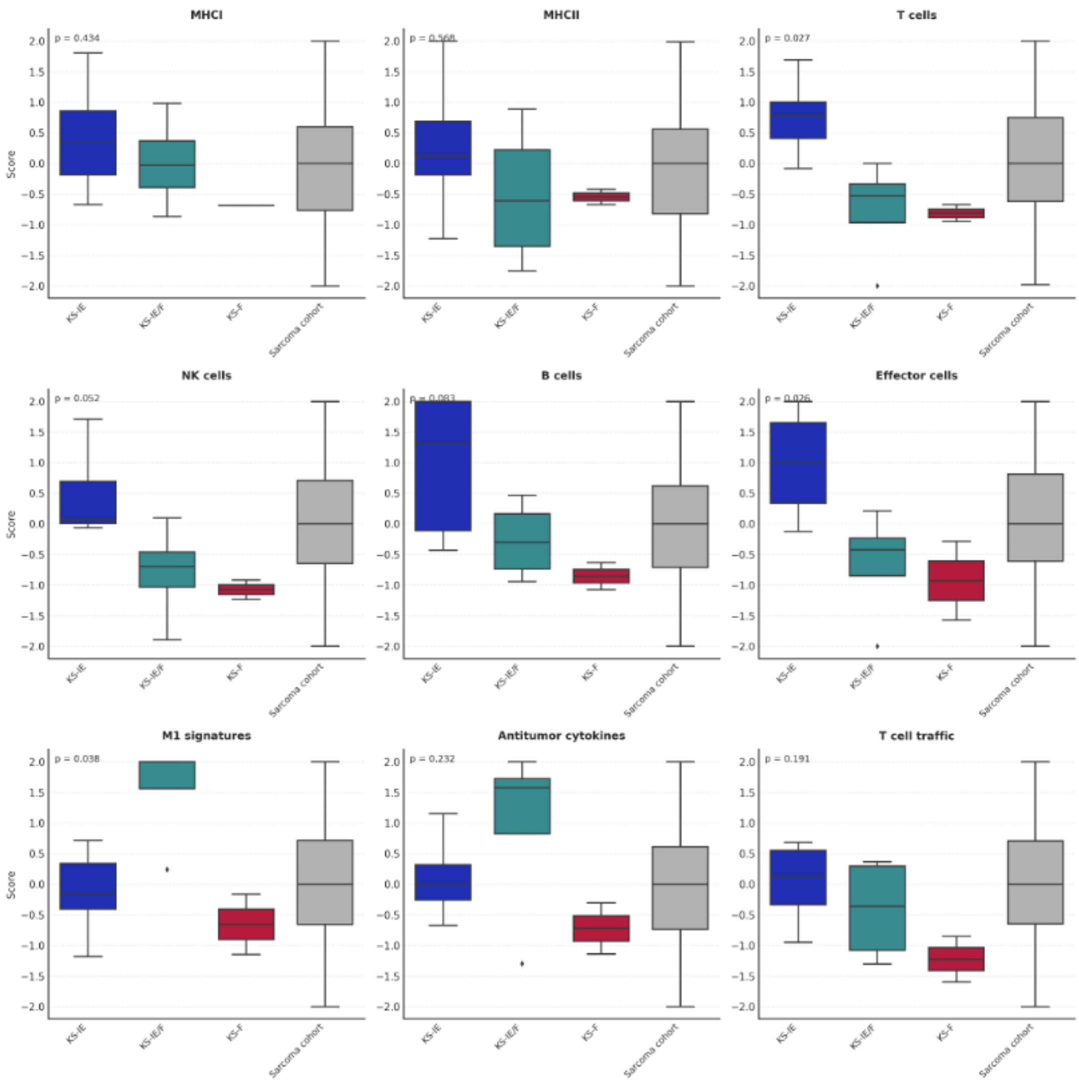

Supplementary Figure S1. Boxplots comparing the immune signature activities of the KS samples to the internal BostonGene sarcoma cohort.
